# An Introduction to the Special Issue on “Challenging Structural Inequities in Education to Promote Positive Psychosocial Outcomes in Migration Diverse Societies”

**DOI:** 10.1002/jcop.70092

**Published:** 2026-02-16

**Authors:** Savaş Karataş, Linda P. Juang, Maja K. Schachner, Tuğçe Aral, Sören Umlauft

**Affiliations:** ^1^ Department of Education and Pedagogy, Educational Psychology – Socialization and Culture Research Group Martin Luther University Halle‐Wittenberg Halle (Saale) Germany; ^2^ College of Human Sciences, Inclusive Education University of Potsdam Potsdam Germany

**Keywords:** education, immigrant descent and minoritized youth, inequities

## Abstract

Global migration has made schools more diverse, yet many immigrant and other marginalized students continue to face unequal educational opportunities. These inequalities are shaped by factors such as socioeconomic disadvantage, limited inclusion efforts, and discriminatory experiences. This special issue brings together 14 studies from seven countries to explore how educational institutions in migration‐diverse societies can reduce these inequalities by better understanding school experiences on multiple levels, including: teachers' beliefs and classroom practices, students' and parents' experiences of school, school climate, curriculum content, and programs designed to support students' identities, sense of belonging, and well‐being. Using a range of methods and educational settings, the studies in this special issue highlight the importance of contextualizing findings within the specific sociohistorical setting, moving beyond deficit‐focused explanations, and supporting inclusive, reflective educational practices to promote more equitable outcomes for all students.

The global increase in migration requires schools to adapt to shifting demographics to ensure educational equity for student populations that are diverse along many dimensions–cultural, racial, ethnic, socioeconomic, and linguistic (Banks 2024). Educational equity exists when all students, including those from minoritized and marginalized groups, are achieving at the same level as their peers belonging to more privileged groups. Educational inequities, however, persist around the globe. This persistence can be partly attributable to the continued limiting of opportunities where immigrant and other minoritized groups are more likely to experience lower socioeconomic resources, encounter language barriers, and be placed in non‐academic school tracks at young ages compared to their non‐immigrant peers, all of which may end up reinforcing negative stereotypes and stigmatization of immigrant and minoritized groups (Banks 2024). On top of this, the COVID‐19 pandemic has contributed to exacerbating educational inequities (Golden et al. 2023). And in many countries, such as the U.S., Argentina, India, and across Europe, governments have shifted or are shifting into nationalism, authoritarianism, and public negativity toward immigrants and minoritized groups. Publicly depicting immigrants and their descendants as threats to society can perpetuate discriminatory experiences (Eberl et al. 2018), which can negatively affect academic outcomes (Verkuyten et al. 2019), contributing to the persistence or increase in educational inequities. Importantly, the impact of such inequities extends beyond individual‐level consequences; they hinder the potential for upward social mobility and overall well‐being of communities, and are a threat to societal stability and cohesion.

Educational institutions are important places for reflecting on and challenging these inequities. Thus, evidence‐based insights on reducing disparities in schools and other education contexts to enhance academic opportunities for all children are essential. The primary objective of this special issue is to address how best to engage with diversity in educational contexts to diminish structural inequities and, ultimately, their influences on educational experiences and psychosocial outcomes of children and youth, but also their parents and educators in migration‐diverse societies.

To address the aims of this special issue, the collection of 14 empirical contributions presents results from samples originating in seven countries, with several of these studies taking a comparative perspective, and also includes a scoping review paper spanning across 12 countries (Özdemir et al. 2026). The studies are also diverse in terms of sample (students, parents, teachers, and curriculum), age range (covering students from ages 10 to 24), institutions (primary school to university), methodologies (qualitative, quantitative, experimental, and scoping review), and theoretical frameworks. In concluding the special issue, Phalet (2026) synthesizes findings in light of an integrative framework incorporating ecological, relational, and intersectional approaches. Drawing on these complementary approaches to systemic inequities in education, the commentary highlights implications for future research.

## 1 Adopting Critical Perspectives Across M(ai)cro Contexts

For this special issue, we have, as guest editors and researchers, adopted a critical perspective by considering our own positionalities, which reflect variations (and thus relations to power and privilege) across immigration status, gender, racialized minority and majority identities, socioeconomic background, and sexual orientation. These positionalities affect our understanding of knowledge production in several ways. First, we include studies that use quantitative and qualitative methodologies. We see both methodologies as valid and complementary ways of understanding educational phenomena, and we do not privilege one over the other. Second, we included studies that contextualize their research questions and findings, interpreting results in relation to broader structural inequities in education rather than relying solely on decontextualized comparisons (e.g., reporting group differences without acknowledging systemic conditions). By doing so, we explicitly reject a‐contextual, deficit‐oriented perspectives on immigrant and other minoritized youth. Third, we selected studies that covered multiple actors within educational systems, such as students, parents, teachers, and schools, to highlight that all actors operate within broader social structures characterized by unequal distributions of power and privilege.

Subsequently, this special issue brings attention to how inequitable systems inform educational experiences and manifest in behaviors and practices in the classroom, curriculum, and community (Gorski and Dalton 2020). Schools are institutions embedded in societies that are structured by inequities where harmful ideologies in the macrosystem, such as racism, sexism, and classism, are threaded throughout microsystems (e.g., schools) and are deeply intertwined (Rogers et al. 2021). Similarly, country‐ or societal‐level intergroup ideologies (e.g., assimilation, colourblindness, and multiculturalism) manifest and are threaded through different levels—from national policies to individual attitudes (Guimond et al. 2014). Thus, our efforts to understand what is happening in classrooms among students and teachers, as well as outside the classroom with families and communities, must necessarily take into account these inseparable m(ai)crosystems (Rogers et al. 2021).

Taking a critical, contextually informed perspective is not just important for us as researchers, but also for educational practitioners. Thus, in the next section, we describe a set of studies in the special issue that highlight the importance of critical perspectives of pre‐ and in‐service teachers. Then, based on Spencer et al.'s (1997) Phenomenological Variant of Ecological Systems Theory (PVEST) framework, we describe a set of studies focusing on how students, parents, and teachers perceive, make sense of, and respond to their contexts. Finally, using Graham's (2018) ethnic diversity framework, the last set of studies provides examples of how contexts and students' outcomes are linked through social processes such as social identities and friendships. Together, these studies suggest that critical and contextualized perspectives are necessary in understanding students' experiences and outcomes.

## 2 Critical Perspectives of Teachers

Four studies in the special issue highlight power relations and dynamics, particularly surrounding teachers who contribute to more or less equitable conditions for students. Two studies focus on pre‐service teachers. Güleç et al.'s (2026) mixed‐methods study examined whether some German pre‐service teachers (e.g., those who experience discrimination or are from lower SES backgrounds themselves) have a more critical perspective regarding educational inequity of students to acknowledge the importance of parental and structural factors beyond individual‐level attributions. This study investigates the important construct of critical consciousness—how people (in this case, future teachers) reflect on and become aware of societal power disparities that ultimately might prompt them to act to reduce educational disparities. Civitillo et al.'s (2025) two‐study paper uses an experimental method to focus on microaggressions, defined as biased, everyday, often subtle interactions targeting racially and ethnically minoritized individuals. The study tested whether German pre‐service teacher characteristics, such as being prejudiced, critically reflective, or having greater empathy, related to their skill at identifying and interpreting microaggressions in the classroom. A meta‐analysis of teacher‐based discrimination shows it is related to poorer academic performance and well‐being among students (Civitillo et al. 2023). Thus, microaggressions are important to focus on as these are subtle and sometimes unintentional discriminatory behaviors that we may not be aware of.

Two studies turn to trainings for in‐service teachers. Gleason et al.'s (2026) interview study reports on an anti‐oppression training for faculty at a predominantly white university in the U.S. Importantly, the training aimed to help educators from diverse disciplines critically examine social power in academic contexts and identify ways to change oppressive practices in their university classrooms. Both critical analysis and building community were emphasized. Similarly, Umaña‐Taylor et al.'s (2025) study of U.S. in‐service teachers describes how they were trained to engage in a critical understanding of how student and teacher identities are intertwined with sociocultural and historical contexts that are inequitable, with implications for student learning. This training had a unique practical component where teachers implemented an Identity Project with their students, so both students and teachers were critically reflecting on identity, power, and learning. All four of these studies adopt critical perspectives on educational inequity, examining how pre‐ and in‐service teachers develop the awareness and skills needed to recognize structural forces and power dynamics that inform students' experiences.

## 3 Perceptions and Meaning Making of M(ai)cro Contexts

Individuals, of course, experience their worlds in their own ways. Spencer and colleagues' Phenomenological Variant of Ecological Systems Theory (PVEST, Spencer et al. 1997) highlights the importance of adolescents' perceptions of their context to make meaning of their experiences. The PVEST framework was meant to highlight the person in Bronfenbrenner's (1979b) ecological systems model to emphasize how the same context can be experienced very differently depending on how each person understands, for instance, societal expectations and stereotypes that are rooted in their lived experiences. The theorizing in PVEST offered important insights into why youth racialized as Black may experience the same academic context differently from youth racialized as white, which then could partly explain academic disparities between these two groups. In line with a PVEST framework, the special issue includes studies that highlight the perceptions of contexts by students, teachers, parents, or all three.

Regarding students, Boer et al. (2025) conducted an experimental study to examine primary school students' expectations of teachers' behavior toward classmates from different socioeconomic and migration backgrounds in the Netherlands. The study's experimental approach provides insights into how children perceive subtle social distinctions based on SES and migration in the classroom, reflecting an awareness of social stratification. Çolak et al.'s (2026) interview study explored how racialized students at an urban secondary school in the Netherlands perceived their interactions with their teachers. Their study highlights the importance of hearing from students themselves about their perceptions of their teachers' pedagogical practices that communicate care and affirmation or dismissiveness and exclusion, which then defines the context that either promotes or weakens a sense of belonging. The authors highlight the need to develop teachers' critical emotional literacy and praxis.

In addition to the school microsystem, Benbow et al.'s (2026) survey study in the U.K. and Germany examined perceptions of the school diversity climate by ethnic/racially minoritized and majoritized parents of school‐aged children and whether these perceptions are related to their school involvement. The study extends the family–school cooperation literature by introducing and investigating the role of cultural diversity and discrimination‐related school efforts in these cooperations. The study also highlights the importance of understanding how parents perceive their children's schools as supportive (a contextual protective factor) or discriminatory (a contextual risk factor), as it can make a difference for their decision to be involved with the school or not.

Taking a mixed‐method approach, Paizan et al.'s (2026) study included students, parents and teachers in Germany and assessed their perceptions of the frequency and quality of home‐to‐school contact and how this was associated with students' sense of school belonging. Importantly, they also considered how these associations may vary by neighborhood SES. In other words, the neighborhoods in which students and their families live may also contribute to how they make meaning of home–school connections. Their study highlights the importance of diversity‐related practices and engagement in schools as important points for intervention to break the link between structural disparities and inequities in educational outcomes.

From a PVEST framework, these four studies collectively show how students' outcomes must be understood by how these contexts are perceived and interpreted. Both students and families interpret school signals (by teachers, a school's approach to diversity) that communicate inclusion or discrimination or both. This then contributes to perceptions of developmental contexts that are more or less supportive for students' academic and socioemotional outcomes.

## 4 Linking M(ai)cro Contexts to Processes and Outcomes

Zooming in on the school context, Graham's (2018) framework on the implications of structural diversity in education provides a useful way to understand how contexts such as those described above are linked to student outcomes through several processes. In this framework, Graham (2018) proposes that the ethnic diversity *context*, such as the density and proportion of ethnically diverse students in classrooms and schools, informs *social processes*. These processes include opportunities and exposure to diverse peers, which may lay the foundations for developing cross‐ethnic friendships, complex social identities, and more positive school conditions where students feel safe and report lower peer victimization. These positive social processes then predict *outcomes*, including socioemotional and academic adjustment.

Regarding *contexts*, classrooms, schools, and neighborhoods provide varying levels of diversity. Consistent with the main tenets of the intergroup contact theory (Allport 1954), growing up in diverse contexts offers multiple opportunities for contact with diverse peers, which, importantly, is linked to lower prejudice against more stigmatized groups (for a meta‐analysis, Raabe and Beelman 2011). Beyond ethnic density, how this diversity is approached, such as manifested in the diversity climate, is important to consider (Schachner et al. 2016) as diversity climate shapes the quality of contact (Karataş et al. 2023), as well as academic and socioemotional outcomes (for a meta‐analysis, see Bardach et al. 2024). Two of the studies in this special issue focus on the school diversity climate, as perceived by students (Vietze et al. 2026) and parents (Benbow et al. 2026, described above). Vietze et al.'s (2026) study of Dutch high school students explored how school‐level diversity approaches (color‐evasion, multiculturalism, and critical consciousness) may relate to student school belonging, well‐being, and intercultural contact. Going beyond direct effects, they also then tested whether classroom‐level climate, defined by perceived openness and appreciation of diversity among classmates with their peers, moderated school‐level diversity approaches with student outcomes. The study highlights how capturing context on multiple levels offers a more specific understanding of how each may contribute uniquely and together to student experiences.

Another important aspect of the educational context is the materials that teachers use, such as textbooks. Jehle et al.'s (2026) comparative school textbook (math and language subjects) analysis focused on ethnic and gender representation in the textbooks of three European countries (i.e., Germany, Italy, and the Netherlands), with a novel/rare focus on intersections, namely the representation of female ethnically minoritized characters. Coding and analysis aimed for tentative comparisons between countries and subjects regarding biased representation of ethnically minoritized and female characters that may align with negative stereotypes of these groups—a so‐called hidden curriculum in schooling materials. This type of analysis is important as textbooks reflect what is considered core knowledge young people should acquire, and also convey societal norms, values, and expectations.

Regarding social processes, Graham's (2018) framework highlights social identities and friendships as key mechanisms linking context to outcomes. Studies in this special issue focus on important social identities such as those related to heritage culture and mainstream culture. In Hillekens et al.'s (2026) study of minoritized early adolescents in Belgium, they focus on whether students' heritage and mainstream cultural identities were linked to the number of friendships. The authors investigate how the particular context of minority‐only schools, where exposure to majority peers is limited, has important implications for social processes such as the development of social identities (e.g., how they identify with their heritage culture and mainstream identities) and how this, in turn, can predict friendships, as Graham (2018) proposes in her model.

Recognizing the importance of social identities, two additional studies test identity interventions among students. In a quasi‐experimental design, Mocevic and Gniewosz (2026) explored the detrimental effects of stereotype threat on academic performance among students of immigrant descent in Austria and evaluated the effectiveness of a multiple (vs. simple) identity intervention in mitigating these effects. The authors argue for attention to the central role of identity for understanding why adolescents may do worse or better in school—better when social identities are affirmed and valued, especially for those whose identities are threatened by being a member of a negatively stereotyped and stigmatized group. Similarly, Pevec‐Zimmer et al. (2025) tested an identity intervention that was meant to promote exploration of ethnic and national identity among ethnic minoritized and majoritized youth in Germany. In contrast to Mocevic et al. they study a different process that may ultimately link social identities to student academic performance—through global identity coherence. In other words, they tested if the intervention promotes exploration of social identities, such as ethnic and national, it may lead to a better overall understanding of the self, which then may be linked to better student outcomes.

Moving to the far side of Graham's (2018) model, regarding outcomes that are linked to social processes (e.g., social identities and friendships), it is proposed that socioemotional and psychological health, as well as academic well‐being, are intertwined. The studies in this special issue cover these various outcomes. Kim et al.'s (2025) longitudinal study of Mexican American youth examines how discrimination by teachers and peers during middle school has long‐term consequences, by influencing educational expectations in young adulthood. While Graham's model focuses only on the school context, these researchers also brought in the family context as an additional micro‐system. The authors tested whether family experiences such as language brokering (e.g., translating and communicating for their immigrant parents) would dampen or exacerbate these links. The study shows how family dynamics can be an additional risk or protective factor altering discrimination effects on educational aspirations specifically.

Özdemir et al.'s (2026) scoping review explores the effects of school‐based interventions designed to support newcomer immigrant youth (living in the resettlement country for up to 5 years) aged 12–15 years. The review of quantitative studies includes 17 interventions from various countries (mostly from North America and Europe, but also including from Australia, India, Lebanon, and Turkey), with most focusing on socioemotional adjustment and strengthening social support. The review emphasizes the importance of considering a broad range of outcomes to understand how youth are doing in school. We also note that while academic and socioemotional outcomes are portrayed as downstream effects in Graham's (2018) framework, they also could reciprocate. Students who feel and do better may also develop broader friendship networks and strengthen their social identities.

## 5 Conclusion

This special issue highlights how structural inequities, social contexts (i.e., school, family, and neighborhood), and individual experiences all contribute to educational outcomes for students in migration‐diverse societies. While systemic factors such as socioeconomic disparities and exclusionary school climates can be considered as contributing to educational inequities, the contributions of this special issue also point to avenues for further investigation of potential protective factors and strengths that can counter these inequities. For instance, future studies could continue to focus on teachers' critical consciousness and reflective practice, interventions that affirm students' social identities, inclusive school diversity climates, positive home–school connections, and curricula that represent diverse identities. These factors show that students, families, and educators can actively shape environments to confront inequities and promote a sense of belonging and more positive school experiences for all youth. Altogether, using a range of methods and educational settings, contributions in this special issue offer a fine‐grained understanding of contextually‐grounded, reflective, and sustainable educational practices to promote more equitable student outcomes, while setting an agenda for future research.

## Conflicts of Interest

The authors declare no conflicts of interest.

## Peer Review

The peer review history for this article is available at https://www.webofscience.com/api/gateway/wos/peer-review/10.1002/jcop.70092.

## Data Availability

Data sharing is not applicable to this article, as no new data were created or analyzed in this study.
